# DisSim: an online system for exploring significant similar diseases and exhibiting potential therapeutic drugs

**DOI:** 10.1038/srep30024

**Published:** 2016-07-26

**Authors:** Liang Cheng, Yue Jiang, Zhenzhen Wang, Hongbo Shi, Jie Sun, Haixiu Yang, Shuo Zhang, Yang Hu, Meng Zhou

**Affiliations:** 1 College of Bioinformatics Science and Technology, Harbin Medical University, Harbin 150081, PR China; 2 Hospital for Sick Children, Toronto, Canada; 3 School of Management, Harbin University of Commerce, Harbin 150081, PR China; 4 School of Life Science and Technology, Harbin Institute of Technology, Harbin 150081, PR China.

## Abstract

The similarity of pair-wise diseases reveals the molecular relationships between them. For example, similar diseases have the potential to be treated by common therapeutic chemicals (TCs). In this paper, we introduced DisSim, an online system for exploring similar diseases, and comparing corresponding TCs. Currently, *DisSim* implemented five state-of-the-art methods to measure the similarity between Disease Ontology (DO) terms and provide the significance of the similarity score. Furthermore, *DisSim* integrated TCs of diseases from the Comparative Toxicogenomics Database (CTD), which can help to identify potential relationships between TCs and similar diseases. The system can be accessed from http://123.59.132.21:8080/DisSim.

Disease similarity has drawn more and more attention in exploring the molecular mechanisms underlying human complex diseases^1^,^2^,^3^,^4^,^5^. In previous studies, Wang *et al.*^3^ and Zeng *et al.*^4^ exploited the similarity between diseases to infer human microRNA function network, and Suthram *et al.* utilized disease similarity to find gene functional modules representing a common disease-state signature^2^. More recently, we used similar diseases to explore potential therapeutic chemicals (TCs) of diseases^1^.

Resources for calculating disease similarity include Online Mendelian Inheritance in Man (OMIM)^6^, Medical Subject Headings (MeSH)^7^, and Disease Ontology (DO)^8^. Among these three resources, OMIM records genetic disorders without providing semantic associations between them. MeSH is a more comprehensive resources for describing disease terms and contains 16 categories, of which only C and F03 involve disease concept. In comparison with OMIM and MeSH, DO has been established around the concept of disease, and it aims to provide a clear definition for each disease. DO provides a more complete and comprehensive description of disease terms. Therefore, a considerable interest is presently attracted to the calculation of similarity between DO terms^1^,^9^,^10^.

The existing approaches for computing the similarity between DO terms often focus on semantic association between diseases and functional association between genes. Among these typical methods, Resnik’s and Lin’s methods^11^,^12^ are based on information content for computing similarity between terms of ontology, and Wang’s method^13^ is based on hierarchical structure of the ontology. These semantic-based methods have served to calculate the similarity of DO terms^14^. More recently, two new methods introduced functional association between genes to improve the performance. One is process-similarity based (PSB) method^10^ which exploited the association of biological process between genes to calculate disease similarity. Another is SemFunSim^1^ which considered more types of the functional association including protein-protein interaction, human mRNA co-expression, and so on. In addition, advanced methods for calculating similarities between diseases of additional resources were also presented. For example, Allan *et al.*^15^,^16^ exploited Jaccard-based similarity index to compare pair-wise diseases of Comparative Toxicogenomics Database (CTD)^17^ based on the number of their shared genes.

In the previous study, DOSim was presented for calculating the similarity between DO terms^14^, which implemented multiple semantic-based methods in an R package. However, it doesn’t provide the significance of the similarity score. In this paper, we presented an online tool *DisSim* to compute the similarity between DO terms, which provides both semantic-based and functional-based methods (Resnik, Lin, Wang, PSB, and SemFunSim). In addition, the system obtains the P-value of the similarity score to provide the significance of it. Furthermore, *DisSim* compares TCs of similar diseases. The system is freely available at http://123.59.132.21:8080/DisSim.

## Results

Two tools including SimDisExplore and SimPDExplore are provided in *DisSim*. The details about the usage of these two tools are described as follows.

### A case for using SimDisExplore

**Figure 1:**
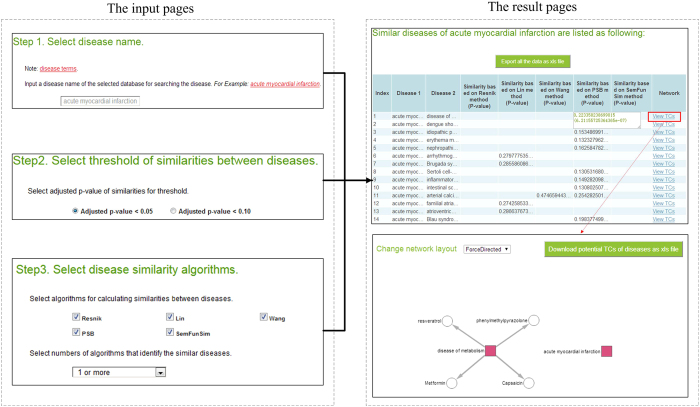
Schematic workflow of SimDisExplore.

SimDisExplore can be accessed from the web http://123.59.132.21:8080/DisSim/single.jsp. Figure 1 shows a case for searching similar diseases of ‘acute myocardial infraction’ and finding correlated TCs of ‘acute myocardial infraction’ and its similar diseases.

#### Searching similar diseases of ‘acute myocardial infraction’

Step 1: Input a disease term. Disease terms of Disease Ontology (DO) in *DisSim* (see Materials and Methods) can be used as reference when inputting a disease term. These disease terms can be downloaded from the page. For convenience, we also provide the function to autocomplete the disease term. In this case, we explored similar diseases of ‘acute myocardial infraction’.

Step 2: Select a threshold for the similarity score. For an inputted disease term, the system will return its similar diseases with the P-value less than 0.10 or 0.05 according to the user’s selection. In this case, we chose the P-value less than 0.05 as the threshold.

Step 3: Select disease similarity algorithms. For an inputted disease term, five algorithms can be used to explore its similar diseases in *DisSim*. Users can select multiple algorithms as they need, and select the frequency of the pair that has been identified as similar diseases. In this case, all of these five algorithms are selected, and the frequency is selected as ‘1 or more’. It means that similar diseases identified by any one of these five algorithms would be shown.

After submitting the input page, similar diseases of the inputted disease term based on the selected algorithms are listed. The first column represents the number of similar diseases. The second and third columns represent the inputted disease and its similar diseases, respectively. The last column is the link to the network visualization of the relationships among TCs and the pair of diseases in this line. Each of the other columns lists the similarity score based on an algorithm and the P-value of the similarity score. All the results can be downloaded from the page.

In this case, the seventh column of the first line is ‘0.223358238699015 (6.21155725364365e-07)’, which means the similarity score between ‘acute myocardial infraction’ and ‘disease of metabolism’ based on the PSB method is 0.223358238699015 and the P-value of the similarity score is 6.21155725364365e-07. The eighth columns is null, which means the P-value of the similarity score between ‘acute myocardial infraction’ and ‘disease of metabolism’ based on SemFunSim method is more than 0.05.

#### Finding potential TCs of ‘acute myocardial infraction’ and its similar diseases

After clicking the link at the last column of the results page of similar diseases, we can get the result page of network visualization of the relationships among TCs and diseases. In this page, each of the red nodes represents a disease, and each of the white nodes is a chemical. For each TC of a disease, we use an edge to link them in the page. The network showing the connections between TCs and diseases is visualized by the Cytoscape Web plugin^18^. Furthermore, potential TCs of diseases are sorted and can be downloaded from the page.

In this case, we got the network among ‘acute myocardial infraction’, ‘disease of metabolism’ and their TCs. The network shows there are no common TCs between ‘acute myocardial infraction’ and ‘disease of metabolism’. This is because ‘acute myocardial infraction’ didn’t be documented in CTD, and the TCs of this disease could not be exploited from CTD directly. According to our system, potential TCs of ‘acute myocardial infraction’ based on its similar diseases could be sorted and downloaded. ‘milrinone’ was one of the potential TCs predicted by our system, and it was validated for treating ‘acute myocardial infraction’^19^.

### A case for using SimPDExplore

**Figure 2:**
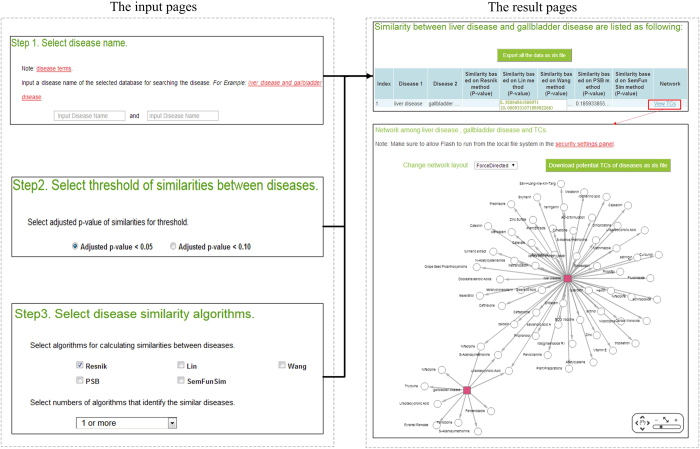
Schematic workflow of SimPDExplore.

SimPDExplore can be accessed from the web (http://123.59.132.21:8080/DisSim/pairs.jsp). Figure 2 shows a case for searching similarity score and finding correlated TCs between ‘liver disease’ and ‘gallbladder disease’. The difference is that SimPDExplore can be used to search similarity of a given pair of diseases.

## Discussion

A recent study showed that similarity of diseases could serve to predict potential TCs of diseases^1^. Although multiple systems have been implemented for calculating the disease similarity, few of them provide potential TCs of diseases and none of them gives significant of the similarity score. In this study, we provided a web interface *DisSim* (http://123.59.132.21:8080/DisSim) for calculating disease similarity based on five state-of-the-art methods and providing the significance of it. Through integrating TCs of diseases from the CTD, *DisSim* can help to identify potential relationships between TCs and similar diseases.

All of these five state-of-the-art methods exploited semantic association of terms in the ontology to calculate disease similarity (see Supplementary Methods). Wang’s method didn’t depend on any other associations. In comparison, Resnik’s and Lin’s methods utilized information content to measure disease similarity, which incorporates the number of disease-related genes. PSB and SemFunSim method further introduced functional associations of genes in the aspects of the biological process and multiple views, respectively.

The advantage of each method mainly depended on the associations they used and the reasonableness of the method. All of these five methods are frequently used and sufficiently verified, which shows that these methods are designed reasonable. Semantic associations of terms are sourced from ontology, which focuses on relationships between terms at the phenotype level and are manually established by domain experts. Therefore, the method based on the semantic associations could be affected by the domain knowledge of experts and the structure of the ontology. In comparison, associations between diseases and genes and functional associations of genes mainly focus on the molecular level. These types of associations could be much more directly but not easy to be identified. In theory, the method based on more types of associations could be more complete and more comprehensive, such as SemFunSim. In contrast, because each type of association is not complete, the method based on fewer types of associations could lead to less bias, such as Wang’s method. In addition, Resnik’s, Lin’s, and PSB methods could be exploited otherwise.

## Materials and Methods

### Data Collection

Data sets of *DisSim* are from open source databases, and they are listed in Table 1. Among them, disease-related genes are from CTD^17^, Gene Reference into Function (GeneRIF)^20^, Online Mendelian Inheritance in Man (OMIM)^6^, Genetic Association Database (GAD)^21^. Disease terms of these databases were assigned to DO according to SIDD^22^. Functional associations between genes are from Gene Ontology (GO), GO Annotation (GOA)^23^,^24^, and HumanNet^25^. And TCs of diseases are from CTD^17^.

### The significance of the similarity score

The existing methods mainly concentrated on calculating the similarity score between DO terms. Few of them provided the significance of the similarity score. In *DisSim*, five state-of-the-art methods including Resnik, Lin, Wang, PSB, and SemFunSim have been put in place for calculating the similarity score between DO terms. For each of these methods, the process of the calculation of the P-value was repeated as follows: 1) First, we calculated similarity scores for all the pairs of diseases; 2) Next, we got the z-score of these similarity scores; 3) Then, one-sided P-value was accessed for each similarity score; 4) Finally, the P-value was adjusted by the BH method^26^. Then, the P-value less than 0.10 or 0.05 is deemed as the threshold of significance.

### The exhibition of potential TCs of diseases

The TCs of diseases from CTD are integrated into DisSim. And the TCs of pair-wise diseases can be compared by the system in order to find the correlated TCs of the disease pair.

Based on comparing TCs of diseases, *DisSim* can provide potential TCs of diseases. According to the hypothesis that similar diseases can be treated by common TCs^2^, TCs of one disease can be used as potential TCs of its similar diseases^1^. For those diseases without TCs in CTD, the TCs of its similar diseases can be easily accessed from DisSim.

### Implementation

**Figure 3:**
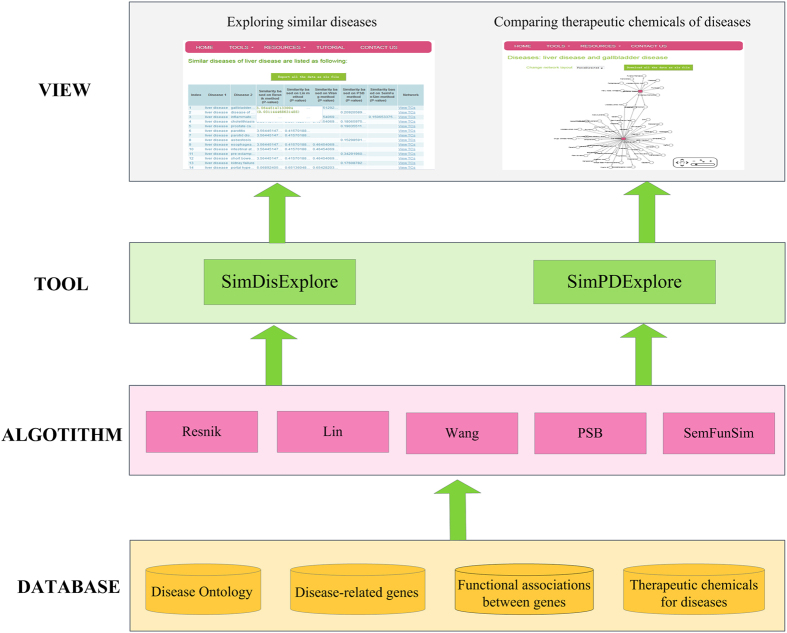
System overview of DisSim.

*DisSim* has been implemented on a JavaEE framework and run on the web server (2-core (2.26 GHz) processors) of UCloud^27^. The four-layer architecture involving DATABASE, ALGORITHM, TOOLS, and VIEW layer is shown in Fig. 3. The detailed description of the architecture is fixed as following.

#### DATABASE layer

This layer stores DO^8^, disease-related genes, functional associations between genes, and therapeutic chemicals (TCs) of diseases. Among them, DO, disease-related genes, and functional associations between genes are exploited by ALGORITHM layer for calculating the similarity between diseases.

#### ALGORITHM layer

Five algorithms of measuring the similarity between DO terms have been implemented, which include Resnik, Lin, Wang, PSB, and SemFunSim.

#### TOOL layer

Two tools including SimDisExplore and SimPDExplore have been provided for exploring the similarity score between diseases. SimDisExplore is used to explore similar diseases for an inputted disease term. In comparison, SimPDExplore calculates the similarity for a given pair of diseases. Both SimDisExplore and SimPDExplore provide the function for comparing TCs of diseases.

#### VIEW layer

Web pages are provided for viewing the results. It shows the similarity of pair-wise diseases and the p-value of the similarity score. It also provides network visualization of relationships among TCs and a pair of similar diseases.

## Additional Information

**How to cite this article**: Cheng, L. *et al.* DisSim: an online system for exploring significant similar diseases and exhibiting potential therapeutic drugs. *Sci. Rep.* **6**, 30024; doi: 10.1038/srep30024 (2016).

## Supplementary Material

Supplementary Information

## Figures and Tables

**Table 1:** Data sources used for measuring disease similarity.

Data source	Web sites for downloading (Date)
DO	http://disease-ontology.org/ (Jun 2016)
CTD	http://ctdbase.org/ (Jun 2016)
GeneRIF	http://www.ncbi.nlm.nih.gov/gene/about-generif (Jun 2016)
GAD	https://geneticassociationdb.nih.gov/(Jun 2016)
OMIM	http://www.omim.org/(Jun 2016)
GO & GOA	http://www.geneontology.org (Jun 2016)
HumanNet	http://www.functionalnet.org/humannet (Jun 2016)
