# Supplementary material for: DisSim: an online system for exploring significant similar diseases and exhibiting potential therapeutic drugs
Source: Sci Rep. 2016 Jul 26;6:30024. doi: 10.1038/srep30024 (PMC4960572; doi:10.1038/srep30024)
Supplement: Supplementary Information [file srep30024-s1.doc]

# DisSim: an online system for exploring significant similar diseases and exhibiting potential therapeutic drugs

Liang Cheng^1§^, Yue Jiang^2^, Zhenzhen Wang^1^, Hongbo Shi^1^, Jie Sun^1^, Haixiu Yang^1^, Shuo Zhang^3^, Yang Hu^4§^, Meng Zhou^1§^

^1^College of Bioinformatics Science and Technology, Harbin Medical University.

^2^Hospital for Sick Children, Toronto.

^3^School of Management, Harbin University of Commerce.

^4^School of Life Science and Technology, Harbin Institute of Technology.

^§^Corresponding author

Liang Cheng, [liangcheng@hrbmu.edu.cn](mailto:liangcheng@hrbmu.edu.cn)

College of Bioinformatics Science and Technology, Harbin Medical University, Harbin 150081, PR China

Yang Hu, [huyang@hit.edu.cn](mailto:huyang@hit.edu.cn)

School of Life Science and Technology, Harbin Institute of Technology, Harbin 150001, PR China

Meng Zhou, [biofomeng@hotmail.com](mailto:biofomeng@hotmail.com)

College of Bioinformatics Science and Technology, Harbin Medical University, Harbin 150081, PR China

# Supplementary Methods

## Resnik’s method [^1^](#_ENREF_1)

The following equation gives the information content of a disease term *t*.

$\text{IC(t)=-log}_{2}\frac{n_{t}}{N},$ (S1)

where *N* denotes the number of different genes related with all the diseases, *N_t_* represents the number of different genes related with *t*, *IC(t)* is the information content of *t*. Then, the similarity of a pair of diseases *t_1_* and *t_2_* by Resnik’s method is defined as the information content of the most informative common ancestor (MICA) of the two diseases, which is described in the equation S2.

$\text{Sim}_{\mathrm{Re}snik}(t_{1},t_{2})\text{=}IC(t_{MICA}).$ (S2)

## Lin’s method [^2^](#_ENREF_2)

According to Lin’s method, similarity of a pair of diseases *t_1_* and *t_2_* could be affected by both MICA of the diseases and information content of each disease, which is defined in the equation S3.

$\text{Sim}_{Lin}(t_{1},t_{2})\text{=}\frac{2\cdot IC(t_{MICA})}{IC(t_{1})\text{+}IC(t_{2})}.$ (S3)

## Wang’s method [^3^](#_ENREF_3)

Assuming *T_1_* is the set involving *t_1_* and all of its ancestor terms of ontology. Semantic contribution of term *t* to *t_1_* could be represented as following:

$S_{\text{t}_{1}}(t)=\left\{\begin{matrix} \text{1 t=t}_{1} \\ S_{\text{t}_{1}}(t)\text{=max\{w}\cdot S_{\text{t}_{1}}(t') | t' \in T_{1} \text{\} t}\neq\text{t}_{1} \end{matrix}\right.\text{，}$ (S3)

where *w* is semantic contribution factor of semantic association. According to Wang et al. [^4^](#_ENREF_4) and Cheng et al [^5^](#_ENREF_5), *w* is defined as 0.5 for ‘IS_A’ relationship of Disease Ontology (DO) [^6^](#_ENREF_6). Then, all the semantic contributions of *T_1_* to *t_1_* is *SV(t_1_)*, which is defined as following:

$S\text{V}(t_{1})=\sum_{\text{t}\in T_{1}} S_{t_{1}}(t).$ (S4)

Assuming *T_2_* is the set involving *t_2_* and all of its ancestor terms, the similarity between *t_1_* and *t_2_* is defined as following by Wang’s method [^3^](#_ENREF_3):

$\text{Sim}_{\text{Wang}}(t_{1},t_{2})\text{=}\frac{\sum_{t\in T_{1}\cap T_{2}} (S_{\text{t}_{1}}(t)+S_{t_{2}}(t))}{SV(t_{1})+SV(t_{2})}$ (S5)

## PSB method [^7^](#_ENREF_7)

Besides semantic associations of DO and associations between diseases and genes, PSB method takes the similarity between disease-related biological processes of Gene Ontology (GO) [^8^](#_ENREF_8) into consideration. For each disease, significant related processes of diseases were identified by hypergeometric test first. Assuming *t_1_* and *t_2_* can be associated with *m* and *n* processes, respectively, the similarity of *t_1_* and *t_2_* is defined as following:

$\text{Sim}_{\text{PSB}}(t_{1},t_{2})\text{=}\frac{1}{2}(\frac{\sum_{\text{i}=1}^{m} \mathop{\text{max}}\limits_{1\leq j\leq n} \text{(}Sim(p_{1i},p_{2j})\text{)}}{m}+\frac{\sum_{\text{j}=1}^{n} \mathop{\text{max}}\limits_{1\leq i\leq m} \text{(}Sim(p_{2j},p_{1i})\text{)}}{n})\text{，}$ (S6)

where $Sim(p_{1i},p_{2j})$ represents similarity between two processes *p_1i_* and *p_2j_*. The similarity between two processes *p_1_* and *p_2_* is described as following:

$Sim(p_{1},p_{2})\text{=}\frac{1}{2}\cdot(IC_{GO}(p_{1})+IC_{GO}(p_{2}))\cdot\frac{n(p_{1}\cap p_{2})}{n(p_{1}\cup p_{2})}\cdot\frac{IC_{GO}(p_{1})}{Max(IC_{GO})}\cdot\frac{IC_{DO}(p_{1})}{Max(IC_{DO})}\cdot\frac{IC_{GO}(p_{2})}{Max(IC_{GO})}\cdot\frac{IC_{DO}(p_{2})}{Max(IC_{DO})},$ (S7)

where $IC_{GO}$ and $IC_{DO}$ represent information content based on GO and DO, respectively. $n(p_{1}\cap p_{2})$ and $n(p_{1}\cup p_{2})$ denote the number of common genes of *p_1_* and *p_2_*, and the number of total genes of *p_1_* and *p_2_*, respectively.

## SemFunSim method [^5^](#_ENREF_5)

Assuming *G_1_* and *G_2_* represent related gene sets of *t_1_* and *t_2_*, respectively. Then, the similarity between *t_1_* and *t_2_* by SemFunSim can be described as following:

$\text{Sim}_{\text{SemFunSim}}(t_{1},t_{2})\text{=}\frac{\sum_{\text{i}=1}^{m} \mathop{\text{max}}\limits_{1\leq j\leq n} \text{(}Sim(g_{1i},g_{2j})\text{)}+\sum_{j=1}^{n} \mathop{\text{max}}\limits_{1\leq i\leq m} \text{(}Sim(g_{2j},g_{1i})\text{)}}{m+n}\cdot\frac{\text{m}}{|G_{MICA}|}\cdot\frac{n}{|G_{MICA}|}\text{，}$ (S8)

where $|G_{MICA}|$ represents the number of genes in $G_{MICA}$. *m* and *n* denote the number of genes in *G_1_* and *G_2_*, respectively. $Sim(g_{1i},g_{2j})$ is the functional similarity score between genes *g_1i_* and *g_2j_*, which could be accessed from HumanNet [^9^](#_ENREF_9).

# References

1. Resnik, P. Using information content to evaluate semantic similarity in a taxonomy. *arXiv preprint cmp-lg/9511007* (1995).

2. Lin, D. An information-theoretic definition of similarity. in *ICML* Vol. 98 296-304 (1998).

3. Wang, J.Z., Du, Z., Payattakool, R., Yu, P.S. & Chen, C.F. A new method to measure the semantic similarity of GO terms. *Bioinformatics* **23**, 1274-81 (2007).

4. Wang, D., Wang, J., Lu, M., Song, F. & Cui, Q. Inferring the human microRNA functional similarity and functional network based on microRNA-associated diseases. *Bioinformatics* **26**, 1644-50 (2010).

5. Cheng, L., Li, J., Ju, P., Peng, J. & Wang, Y. SemFunSim: a new method for measuring disease similarity by integrating semantic and gene functional association. *PLoS One* **9**, e99415 (2014).

6. Schriml, L.M. *et al.* Disease Ontology: a backbone for disease semantic integration. *Nucleic Acids Res* **40**, D940-6 (2012).

7. Mathur, S. & Dinakarpandian, D. Finding disease similarity based on implicit semantic similarity. *J Biomed Inform* **45**, 363-71 (2012).

8. Ashburner, M. *et al.* Gene ontology: tool for the unification of biology. The Gene Ontology Consortium. *Nat Genet* **25**, 25-9 (2000).

9. Lee, I., Blom, U.M., Wang, P.I., Shim, J.E. & Marcotte, E.M. Prioritizing candidate disease genes by network-based boosting of genome-wide association data. *Genome Res* **21**, 1109-21 (2011).
